# Reversing Antibiotic Resistance: Strategies From Adjuvants to Innovative Therapeutics

**DOI:** 10.1002/mbo3.70233

**Published:** 2026-02-15

**Authors:** Tianjiao Li, Fei Zeng, Jie Zhang, Yuangong Zhang, Wenjuan Yin

**Affiliations:** ^1^ College of Basic Medical Science, Key Laboratory of Pathogenesis Mechanism and Control of Inflammatory‐Autoimmune Diseases of Hebei Province Hebei University Baoding China

**Keywords:** antibiotic adjuvants, antimicrobial resistance, innovative strategies, molecular mechanisms, reverse resistance

## Abstract

The escalating prevalence of antibiotic resistance has become a major threat to the effectiveness of conventional antibiotics. Meanwhile, the development of novel antibiotics faces substantial challenges, including lengthy research cycles, high costs, and the rapid emergence of bacterial tolerance, making it difficult for new drugs to keep pace with bacterial evolution. In this context, molecular reversal strategies targeting antibiotic resistance genes have emerged as a promising avenue to overcome this impasse. Among them, the use of antibiotic adjuvants, agents that enhance the efficacy of existing antibiotics by inhibiting resistance gene function, preventing their horizontal transfer or modulating host defense has gained considerable attention. Furthermore, innovative approaches such as CRISPR‐Cas gene editing, photodynamic therapy, nanotechnology, and ecological competition strategies have shown great potential in reversing antimicrobial resistance. Collectively, these strategies offer novel insights into addressing the global crisis of antibiotic resistance, paving the way for more effective clinical interventions and ensuring the sustained efficacy of current antibiotic therapies.

## Graphical abstract

Molecular reversal strategies against antibiotic resistance act through three mechanisms: inhibiting resistance gene function, blocking horizontal gene transfer, and modulating host defense. Emerging technologies, such as CRISPR‐Cas gene editing, photodynamic therapy, nanotechnology, and ecological competition, further strengthen these approaches, offering innovative solutions to restore antibiotic efficacy.

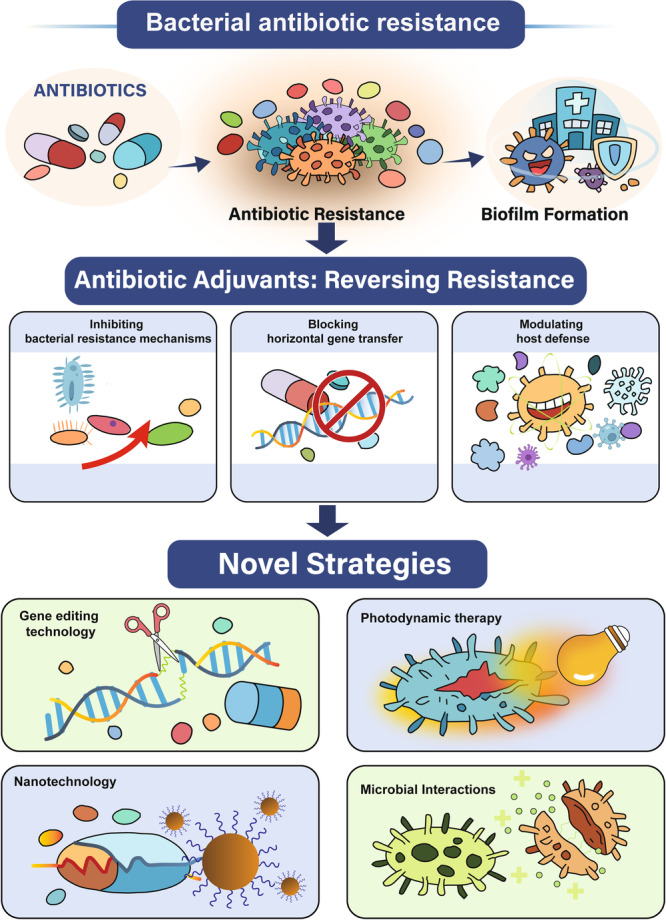

## 1 Introduction

**Figure 1:**
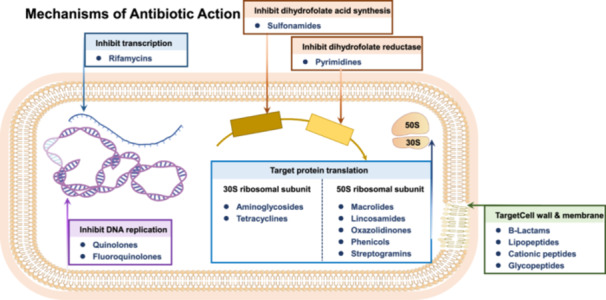
The main types of antibiotics and their mechanisms.

Throughout human history, microbial infections posed significant threats prior to the advent of antibiotics. The emergence of antibiotics revolutionized the treatment of previously life‐threatening bacterial infections, becoming an indispensable pillar of human health. Antibiotics primarily used for the elimination of pathogenic bacteria can be categorized into ten major classes, which, based on their mechanisms of resistance, can be further grouped into five functional types (Boolchandani et al. 2019), as illustrated in Figure 1. Statistically, the use of antimicrobial drugs has extended the average human lifespan by at least 10 years. Despite these remarkable achievements, the issue of bacterial resistance to antibiotics is progressively escalating (Zhuang et al. 2021). Currently, antibiotic resistance has reached a critical global level, continuing to spread and intensify, posing one of the greatest threats to global health, food safety, and development. The resulting problem of antibiotic residues and bacterial resistance burden the entire ecosystem, with antibiotic resistance genes (ARGs) entering the food chain and spreading (Kvesić et al. 2022; Su et al. 2020). The presence of antibiotic‐resistant bacteria (ARB) leads to increased medical costs, along with serious antimicrobial side effects that can significantly harms patients (Sharifzadeh et al. 2021). Antimicrobial resistance (AMR) is a primary cause of mortality in patients with severe infections and is widely recognized as a major threat to human health, posing a formidable challenge to human, animal, and environmental health (Murray et al. 2022). In 2015, approximately 33,000 patients in the European Union and the European Economic Area died due to infections with ARB (Sanganyado and Gwenzi 2019). Projections indicate that by 2050, bacterial antibiotic resistance could lead to the deaths of 10 million people and impose an economic burden of approximately $10 trillion (Nordmann and Poirel 2019). Recognizing the severity of ARB, the World Health Organization (WHO) has listed AMR as one of the top ten global public health threats. Given the current scarcity of effective drug options against superbugs, it is imperative to develop innovative approaches to deal with this threat. Among these strategies, reversing bacterial resistance and restoring susceptibility to antibiotics have garnered significant attention. The objective of this paper is to comprehensively discuss the molecular mechanisms and transmission pathways of bacterial antibiotic resistance, and systematically explore the targets and intervention strategies of antibiotic adjuvants. Additionally, this paper also looks forward novel technologies to advance new strategies that can effectively combat AMR. In this manner, it presents a feasible solution to the clinical treatment challenges posed by AMR.

## 2 Molecular Basis and Dissemination of Antibiotic Resistance

### 2.1 Molecular Mechanisms Underlying Bacterial Antibiotic Resistance

**Figure 2:**
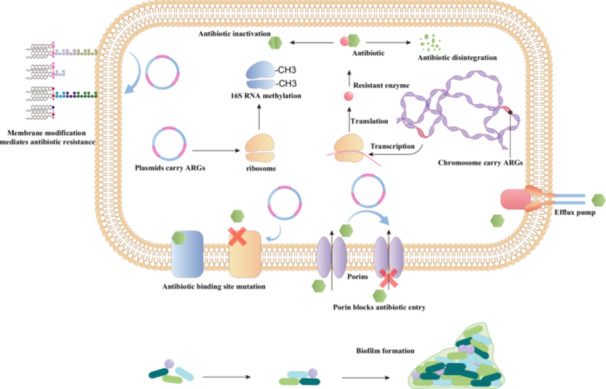
Mechanism of antibiotic resistance in bacteria: ARB acquire resistance phenotypes through multiple mechanisms, including reduced outer membrane permeability, modification of antibiotic targets, porin blockage, overexpression of efflux pumps, enzymatic degradation or modification of antibiotics and biofilm formation.

AMR arises when bacteria acquire or evolve mechanisms that reduce their susceptibility to antibiotics. At the genetic level, AMR originates from mutations, recombination, and horizontal gene transfer events that generate resistant genotypes, which subsequently drive the emergence of complex phenotypes such as multidrug‐resistant (MDR) strains (Pu et al. 2023). Resistant phenotypes manifest through target modification, altered outer membrane permeability, efflux pump overexpression, enzymatic antibiotic inactivation or modification and biofilm formation (Figure 2).

Modification of antibiotic targets represents a major driver of AMR. The *erm* family of genes encodes rRNA methyltransferases that methylate adenine residue within the 23S rRNA, thereby reducing the binding affinity of macrolides, lincosamides and streptogramin B antibiotics to their ribosomal targets (Vimberg et al. 2020). Similarly, mutations in the *gyrA* and *parC* genes alter the quinolone‐binding sites of DNA gyrase and topoisomerase IV, conferring the fluoroquinolone resistance (Wang et al. 2025). In β‐lactam‐resistant pathogens, acquisition of *mecA* leads to expression of PBP2a, a penicillin‐binding protein with markedly reduced affinity for β‐lactams, thereby mediating methicillin resistance in *Staphylococcus aureus* (Lade and Kim 2023). In Gram‐negative bacteria, structural changes in the outer membrane can also confer resistance (Sabnis et al. 2021).

Alterations in membrane permeability constitute another major determinant of AMR. For antibiotics to exert their effect, they must traverse the bacterial envelope to reach intracellular targets. Outer membrane proteins (OMPs) serve as key conduits for antibiotic uptake, and reduced or altered OMP expression, by altering size, abundance, or charge, can hinder drug penetration and confer resistance. Mutations that reduce or eliminate outer membrane porins can severely restrict this influx. For example, the loss of OprD in *Pseudomonas aeruginosa* decreases carbapenem permeability (Li et al. 2012). In *Helicobacter pylori*, mutants lacking *hopE* and/or *hopD* exhibit significantly elevated minimum inhibitory concentrations (MICs) to multiple antibiotics, underscoring the importance of OMPs as molecular conduits for drug entry (Liu et al. 2022).

Efflux pumps are membrane transport proteins that expel diverse substrates to maintain cellular homeostasis and represent a major mechanism of AMR by decreasing intracellular drug accumulation. Prominent examples include the RND‐type MexAB‐OprM and MexXY‐OprM complexes in *P. aeruginosa*, the AcrAB‐TolC system in *E. coli*, and NorA in *S. aureus*, which collectively reduce intracellular drug concentrations and confer cross‐resistance to structurally unrelated antibiotics (Webber 2003). Overexpression of efflux pumps not only diminishes antibiotic efficacy but also facilitates co‐selection of resistance determinants through stress‐induced regulatory networks.

Enzymatic inactivation or modification of antibiotics remains one of the most powerful bacterial defense strategies. Carbapenemases, such as the KPC, NDM and OXA, hydrolyze β‐lactam rings and render antibiotics inactive (Martínez‐Martínez and González‐López 2014). Aminoglycoside resistance is frequently mediated by acetyltransferases, phosphotransferases and nucleotidyl transferases, which covalently modify amino or hydroxyl groups, reducing their affinity for ribosomal binding sites (Band and Weiss 2021). The dissemination of plasmid‐encoded resistance enzymes across bacterial populations has accelerated the global spread of MDR phenotypes, posing severe challenges for clinical therapy.

Biofilm formation provides an additional, multifaceted barrier to antimicrobial penetration. Within biofilms, bacteria are embedded in a matrix of extracellular polysaccharides, proteins, and extracellular DNA, which not only hinders antibiotic diffusion but also establishes chemical and electrostatic gradients that reduce local drug activity (Dieltjens et al. 2020). Furthermore, biofilm‐associated cells often enter a metabolically quiescent state, decreasing the efficacy of antibiotics that target active cellular processes. The dense microbial consortia in biofilms also serve as hotspots for horizontal gene transfer, facilitating the dissemination of resistance determinants.

Diverse antibiotic modes of action have driven the parallel evolution of corresponding resistance mechanisms, posing major therapeutic challenges. Given the difficulty of developing novel agents with new mechanisms and the presence of cross‐resistance among existing drugs, research is increasingly focusing on strategies to reverse these mechanisms. Leveraging current antibiotics alongside innovative interventions can restore efficacy and improve clinical outcomes.

### 2.2 Transfer of ARGs

**Figure 3:**
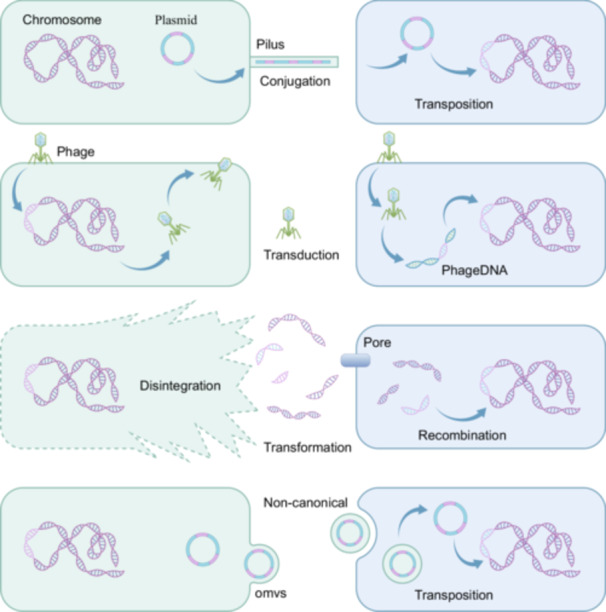
The transfer of ARGs between bacteria: Bacteria can acquire resistant plasmids and ARGs through conjugation, transduction, natural transformation, and transfer of membrane capsules (MVs).

Horizontal transfer of ARGs is a major driver of AMR dissemination in clinical settings (Jian et al. 2021). Horizontal gene transfer (HGT) enables prokaryotes to acquire genetic material from unrelated strains via conjugation, natural transformation, transduction, and membrane vesicle (MV) transmission (Figure 3) (Brito 2021).

Mobile genetic elements (MGEs), including plasmids, integrons and transposons, facilitate HGT between both homologous and heterologous bacteria (Tokuda and Shintani 2024). Plasmids, such as pX3_NDM‐5 carrying carbapenem resistance, can transfer across diverse Gram‐negative species (Yang et al. 2024; dos Santos et al. 2017). Conjugation efficiency depends on bacterial structures and metabolic regulation, with the Type IV secretion system (T4SS) delivering DNA, proteins or other macromolecules to promote ARG transfer (Costa et al. 2021). Transposons insert into diverse genomic sites via transposase or recombinase, driving ARG spread; for example, Tn*6394*, Tn*6375*, Tn*6411* and Tn*6397* mobilize *bla*_IMP_ into plasmids or chromosomes of *Klebsiella pneumoniae*, *P. aeruginosa* and Enterobacter (Rubio‐Cosials et al. 2018; Stalder et al. 2019; Zhan et al. 2018). Integrons capture, rearrange, and express resistance genes, relying on plasmids or transposons for mobility, and are key contributors to MDR (Ghaly et al. 2020).

DNA carrying ARGs can be protected from deoxyribonuclease degradation and persist in the environment long after the original resistant strain has disappeared, enabling acquisition of resistance via natural transformation (Cochran et al. 2024). Phage‐mediated transduction also facilitates DNA transfer, with lateral transduction and cotransduction markedly increasing the frequency of resistance evolution (Chee et al. 2023). Additionally, outer membrane vesicles (OMVs) secreted by Gram‐negative bacteria, such as *E. coli* and *A. baumannii*, can encapsulate and deliver genetic material across species, serving as another route for horizontal gene transfer (Fulsundar et al. 2014).

HGT of ARGs is a key pathway for the transmission of antimicrobial resistance. In‐depth exploration of the fine mechanisms of gene expression regulation during HGT, as well as how intracellular ion concentrations affect coupling efficiency, will provide valuable insights into the underlying inhibition mechanisms.

## 3 Strategies for Reversing Antibiotic Resistance With Antibiotic Adjuvants

The horizontal spread of ARGs has intensified the threat of antibiotic failure in clinical practice. Without effective new drugs, mortality from antibiotic failure will continue to rise. In recent years, no highly effective antibiotics targeting Gram‐negative bacteria have been developed, and only a few candidates have reached Phase II or III clinical trials (Palencia‐Gándara et al. 2021). Most antibiotics were originally derived from metabolites of bacteria, fungi or other microorganisms, but their natural structural diversity is nearly exhausted, creating a bottleneck in novel antibiotic discovery. Moreover, the return on investment is low, as agents requiring 10–15 years of development may lose efficacy within 2–3 years of approval. The WHO Global Health Estimates Report (2020) confirms that current antibacterial needs remain unmet (W.H.O 2019). Addressing this crisis requires targeting its root cause, restoring bacterial susceptibility to existing antibiotics. Among the available strategies, directly targeting bacterial ARGs has proven to be one of the most effective means of reversing resistance.

**Figure 4:**
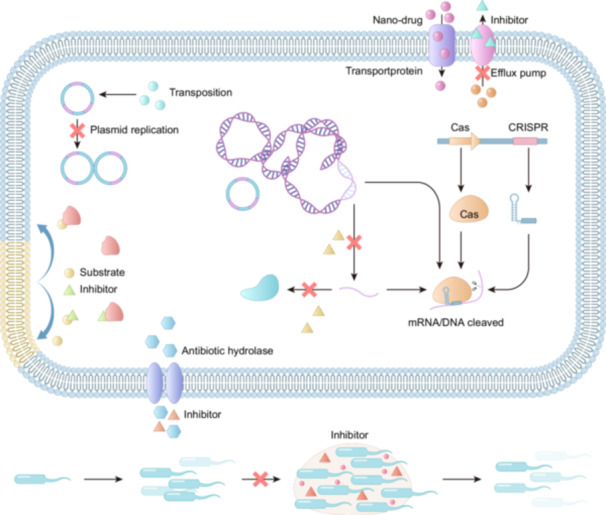
Mechanisms to reverse antibiotic resistance: Pathways to reverse bacterial antibiotic resistance: chemical drugs block the horizontal transfer of ARGs, combination of enzyme inhibitors and antibiotics, non‐antibiotic reverse genetic modification, elimination of biofilms, and gene editing technology targeted elimination of ARGs.

In response to the escalating AMR crisis, antibiotic adjuvants have emerged as an important approach to protect existing antibiotics and treat infections caused by antimicrobial‐resistant bacteria (ARB) (Farha and Brown 2013). These compounds, with little or no intrinsic antibacterial activity, enhance antibiotic efficacy by inhibiting resistance mechanisms or improving antibiotic action (Table 1) (Wright 2016). Based on their mechanisms of action, antibiotic adjuvants are classified into three mechanistic categories. Class I adjuvants block bacterial resistance mechanisms by inhibiting active mechanisms and overcoming metabolic or physiological barriers (Figure 4); Class Ⅱadjuvants prevent the horizontal transfer of ARGs; Class Ⅲ adjuvants potentiate antibiotics via host defense modulation (Farha and Brown 2013; Kumar et al. 2023). Acting synergistically with antibiotics, adjuvants not only improve therapeutic outcomes but also suppress the emergence of resistance, thereby creating a durable defense against AMR (Kumar et al. 2023). Additionally, they can exert additive antibacterial effects by increasing antibiotic‐induced oxidative stress, facilitating biofilm penetration and activating host immunity, thereby delivering benefits across multiple fronts (Feng et al. 2025).

**Table 1:** Classification and representative mechanisms of antibiotic adjuvants.

Class	Mode of action	Target or pathway	Enhanced antibiotic/Therapeutic effect	References
Class I Inhibiting bacterial resistance mechanisms	Efflux pump inhibition	MexAB‐OprM AcrAB‐TolC, MmpS5L5	fluoroquinolone β‐lactam rifampin, tigecycline	(P. Tegos et al. (2011); Douafer et al. (2019); Liu et al. (2023); Moulick and Roy (2024); Chen et al. (2025a); Compagne et al. (2023); Yamasaki et al. (2022); Aron and Opperman (2016); Grossman et al. (2015); Fountain et al. (2025); Duda‐Madej et al. (2025); Kanagaratnam et al. (2017); Zai et al. (2025); Shaheen et al. (2019); Li et al. (2015); Andrade et al. (2017); Liu et al. (2024a); Zheng et al. (2025))
	Enzyme inhibition	β‐lactamases Carbapenemases,	β‐lactam carbapenem	(Bush and Bradford (2019); Agarwal et al. (2023); Drawz and Bonomo (2010); White et al. (2004); Mangat et al. (2019); Castanheira et al. (2021))
	Membrane permeability enhancement	Outer membrane porins, LPS, lipid bilayer	rifampicin colistin	(Yahav et al. (2020); Barnes et al. (2019); Yan et al. (2023); Catteau et al. (2017); Clarke et al. (2025))
	Others	Biofilm inhibition protection of antibiotic targets	β‐lactam	(Ayerbe‐Algaba et al. (2018))
Class II Blocking horizontal gene transfer	Inhibition of horizontal gene transfer (HGT)	Conjugation, fimbriae expression	Reduces ARG dissemination across bacterial species	(Yadav et al. (2020); Ayerbe‐Algaba et al. (2019); Song et al. (2020); Eom et al. (2016))
	Plasmid elimination (curing)	Plasmid replication machinery	Eliminates resistance plasmids, restoring susceptibility	(Chen et al. (2019a); Ahmad et al. (2022); Zhang et al. (2025); Jin et al. (2020); Swain and Sahoo (2025); Nash et al. (2012); Pachnerová Brabcová et al. (2018))
Class III Modulating host defense to enhance antibiotic efficacy	Innate immunity activation	Macrophage activation, Oxidative burst	Enhances host clearance of MRSA and other AMR pathogens	(Popelářová et al. (2022); Vrancianu et al. (2020); Ji et al. (2021); Boy et al. (2022))
	Antimicrobial peptide synergy	Membrane disruption Pore formation	Increases permeability to rifampicin, erythromycin, vancomycin	(Zheng et al. (2025); Ji et al. (2021))

### 3.1 Class I—Inhibiting Bacterial Resistance Mechanisms

#### 3.1.1 Combination of Efflux Pump Inhibition and Antibiotics

Microbial efflux pumps are key drivers of clinically relevant resistance to many antibiotics, thereby contributing to multidrug resistance (MDR). Blocking these promiscuous transport systems with small‐molecule efflux pump inhibitors (EPIs) represents one of the most promising strategies to restore antibiotic susceptibility (P. Tegos et al. 2011). Four mechanistic themes have been identified (Table 2): (a) suppression of efflux pump gene overexpression, leading to fewer active complexes within the bacterial envelope; (b) disruption of pump assembly, preventing functional efflux complex formation; (c) perturbation of membrane or porin architecture, increasing permeability and promoting intracellular drug retention; and (d) depletion of the proton motive force, thereby lowering efflux efficiency (Douafer et al. 2019). By simultaneously “closing the pump, cutting the fuel, opening the gate, and disarming virulence,” EPIs are emerging as central pillars of anti‐MDR therapy and prime candidates for next‐generation antibiotic adjuvants. While, mdiazabicyclooctaneost efflux pump inhibitors (EPIs) remain at the preclinical research stage, with no clinical applications reported to date.

**Table 2:** Efflux pump‐targeting adjuvants and their roles in enhancing antibiotic efficacy against resistant bacteria.

Mechanism	Adjuvant	Targeted efflux pumps	Bacterial strain	Enhanced antibiotic
Inhibition of overexpression of efflux pump genes	Piperine (Liu et al. 2023)	MexAB‐OprM	*P. aeruginosa*	Carbapenem
	Baicalein (Moulick and Roy 2024)	Tet (K), norB	*S. aureus*	Tetracycline
	Quercetin (Chen et al. 2025a)	norA, norB, norC, mepA	*S. aureus*	Quinolones
Blockade of pump assembly	PAβN (Compagne et al. 2023)	Resistance nodulation‐cel division (RND) superfamily	Gram‐Negative Bacteria	Fluoroquinolones, Macrolides, Oxazolidinones, Chloramphenicol, Rifampin
	ABI‐PP (Yamasaki et al. 2022)	MexB and AcrB	*P. aeruginosa* *E. coli*	Erythromycin
	Pyranopyridine (Aron and Opperman 2016)	AcrAB	*E. coli* *S. flexneri* *K. pneumoniae* *S. enterica* serovar Typhimuriam Enterobacter cloacae	Fluoroquinolones, β‐lactams ChloramphenicolMinocycline Erythromycin Linezolid
	Timcodar (Grossman et al. 2015)	P‐gp, BCRP MRP1	*M. tuberculosis*	Rifampin Moxifloxacin Bedaquiline
	Verapamil (Fountain et al. 2025)	MmpS5L5	*M. tuberculosis*	Bedaquiline
	Berberine (Duda‐Madej et al. 2025)	MexAB‐OprM, AdeABC MexXY‐OprM AcrAB‐TolC	*P. aeruginosa*, *A. baumannii* Enterobacteriaceae	Ciprofloxacin Tigecycline Meropenem
	Epigallocatechin‐3‐gallate (Kanagaratnam et al. 2017)	MexAB‐OprM	*P. aeruginosa*	Chloramphenicol Tetracyclines
	luteolin (Zai et al. 2025)	MsrA	*Trueperella pyogenes*	Macrolides
	Reserpin (Shaheen et al. 2019)	AcrB	*M. tuberculosis* *S. aureus*	Ciprofloxacin
Blockade of the membrane outlet duct	Glycine basic peptide (Li et al. 2015)	Leakage of ions Ca^2+^, K^+^, Mg^2+^	*E. coli*	Erythromycin Rifampicin
	Menadione (Andrade et al. 2017)	disturbances in the bacterial membrane	*S. aureus* *P. aeruginosa* *E. coli*	Aminoglycosides
Depletion of the energy	Metformin (Liu et al. 2024a)	TMexCD1‐TOprJ1	*K. pneumoniae*	Tigecycline
	Curcumin (Zheng et al. 2025)	Tet(X4)	*E. coli*	Tigecycline

#### 3.1.2 Combination of Enzyme Inhibitors and Antibiotics

Antibiotic resistance enzyme inhibitors can effectively reduce or inhibit the activity of bacterial‐produced resistance enzymes, and their application can restore the sensitivity of bacteria to drugs and achieve the goal of reversing bacterial resistance. The antibiotic resistance enzyme inhibitors combined with corresponding antibiotics have been widely used in clinical practice, especially in combination with β‐lactam antibiotics and carbapenem antibiotics. The commonly used β‐lactamase inhibitors (BLIs) in clinical practice include: clavulanic acid, sulbactam, tazobactam, avibactam, relebactam, and fobactam (Bush and Bradford 2019). The first three all contain a β‐lactam ring structure, are irreversible competitive inhibitors, and can inhibit most A‐class β‐lactamases except for carbapenemases (Agarwal et al. 2023). The earliest β‐lactamase inhibitors to be described and introduced into clinical use were clavulanic acid and sulbactam (Kumar et al. 2023). Since clavulanic acid was first approved by the U.S. FDA in 1984, a total of seven β‐lactamase inhibitors have been approved for clinical use to date (Drawz and Bonomo 2010). Among them, the combination of amoxicillin and clavulanic acid represents the most widely prescribed oral antibacterial formulation, clinically applied to treat a broad spectrum of infections, including bronchitis as well as skin and soft tissue infections (White et al. 2004).

Avibactam and relebactam are BLIs belonging to the (DABCOs), which do not have a β‐lactam ring structure and are therefore less susceptible to hydrolysis, have a more broad‐spectrum β‐lactamase inhibition and reversible inhibitory effect, and can inhibit A‐class, C‐class β‐lactamases, including carbapenemases (Mangat et al. 2019). Avibactam also has an inhibitory effect on d‐class enzymes, including OXA‐48, but relebactam is unable to inhibit OXA‐48 (Castanheira et al. 2021). Clinically, avibactam was approved in 2015 and is currently co‐formulated with ceftazidime, while vaborbactam, approved in 2017, is combined with meropenem, both showing markedly improved clinical efficacy of β‐lactam antibiotics against multidrug‐resistant (MDR) Gram‐negative pathogens (Yahav et al. 2020). Furthermore, the inhibitor relebactam (MK‐7655) has been approved for use in combination with imipenem/cilastatin (imipenem/cilastatin/relebactam) to combat infections caused by carbapenemase‐producing bacteria that inactivate imipenem (Bush and Bradford 2019; White et al. 2004).

The appearance of BLIs has significantly alleviated the problem of β‐lactam antibiotic resistance. However, currently approved inhibitors still show limited efficacy against class C (AmpC) and class D (OXA‐type) β‐lactamases, which restricts their clinical utility. To overcome this limitation, a novel bicyclic diazabicyclooctanone inhibitor, ETX2514, was developed in 2019 (Barnes et al. 2019). It exhibits potent inhibitory activity against class A, C and D β‐lactamases, and its combination with sulbactam has demonstrated significant clinical efficacy in the treatment of MDR *A. baumannii* infections. There have also been studies that have successfully demonstrated the potential of 2‐amino‐4‐thiazolecarboxylic acid (AtCs) to restore the activity of meropenem against metal‐ β‐lactamases (MBLs) by simulating the binding of carbapenem hydrolysis products (Yan et al. 2023).

Natural products are now entering the same therapeutic space. Ursolic acid and oleanolic acid isolated from shea‐tree leaves inhibit β‐lactamase activity in intact bacteria, while the FDA‐approved combination of resveratrol, eugenol and astragalus with colistin exemplifies how phytochemicals can restore the activity of last‐resort antibiotics against colistin‐resistant Gram‐negative pathogens (Catteau et al. 2017). Together, these synthetic and natural enzyme inhibitors provide an expanding arsenal for combating resistance mediated by β‐lactamases, carbapenemases and MBLs. Collectively, these β‐lactamase inhibitors represent the most clinically validated class of antibiotic adjuvants. Their success demonstrates that targeted inhibition of resistance enzymes can reliably restore the efficacy of frontline β‐lactams, and provides a model for the development of next‐generation adjuvants.

#### 3.1.3 Combination of Membrane Permeability Enhancers and Antibiotics

The efficiency of antibiotic penetration into bacterial cells is closely associated with membrane permeability. Therefore, adjuvants that alter porin expression, membrane lipid composition, or membrane potential can significantly enhance the efficacy of co‐administered antibiotics (Douafer et al. 2019; Clarke et al. 2025). For instance, niclosamide increases the negative surface charge of bacterial membranes, restoring susceptibility to colistin (Ayerbe‐Algaba et al. 2018). Compounds such as water‐soluble curcumin and oxyclozanide enhance membrane permeability and thereby broaden the antibacterial spectrum of partner antibiotics (Yadav et al. 2020; Ayerbe‐Algaba et al. 2019). The short linear antimicrobial peptide (SLAP)‐S25 binds LPS and phosphatidylglycerol, thereby disrupting both outer and plasma membranes and potentiating ofloxacin, rifampicin and vancomycin against MDR Gram‐negative pathogens (Song et al. 2020). Hydrophobic antibiotics (e.g., aminoglycosides and rifampicin) primarily diffuse through the lipid bilayer, whereas hydrophilic agents (e.g., β‐lactams and fluoroquinolones) traverse porin channels; agents such as detergents, surfactants, and antimicrobial peptides can modify these channels to promote antibiotic uptake (Clarke et al. 2025). The glycine basic peptide (GBP) has been shown to increase the permeability of Gram‐negative bacteria, thereby enhancing susceptibility to erythromycin and rifampicin. Moreover, membrane permeability enhancers can be conjugated with efflux pump inhibitors to improve their intracellular delivery, or may intrinsically possess dual functions, enhancing permeability while simultaneously inhibiting efflux, thus providing broad‐spectrum antibiotic potentiation.

In addition to the aforementioned strategies, adjuvants have been engineered to counteract alternative resistance mechanisms, including biofilm inhibition and protection of antibiotic targets. Likewise, Poncirus trifoliata ethyl extract fraction can be inhibited the production of penicillin‐binding protein 2a (PBP2a) in MRSA to eliminate β‐lactam resistance (Eom et al. 2016).

Class I adjuvants act directly on bacterial defense systems by inhibiting efflux pumps, resistance enzymes, permeability barriers, and other resistance mechanisms, thereby restoring antibiotic susceptibility. They represent the most established and mechanistically diverse category of antibiotic adjuvants, offering a practical foundation for combating multidrug resistance.

### 3.2 Class II—Blocking Horizontal Gene Transfer of ARGs

Targeted therapeutics designed to limit the dissemination of ARGs offer an effective strategy to confront the escalating challenge of antibiotic resistance and mitigate the detrimental impacts of ARB (Chen et al. 2019a). The use of conjugative transfer inhibitors to reduce the efficiency of the transfer can be used to reduce the spread of bacterial ARGs. Synthetic fatty acid 2‐cetacic acid, as a conjugation inhibitor, reduced the plasmid coupling frequency both in the controlled water microenvironment and in the mouse intestine (Palencia‐Gándara et al. 2021). Alfalfa oil has the potential to inhibit the transfer of the vancomycin‐resistant *Enterococcus faecium* (VREfm) to methicillin‐resistant *Staphylococcus aureus* (MRSA) and can be used to supplement and enhance the eradication strategy for VRE by using alfalfa oil rich in conjugative transfer inhibitors such as unsaturated fatty acids (Ahmad et al. 2022). Fimbriae play a essential role in plasmid conjugation, and high‐dose chlorine treatment significantly reduces their abundance, thereby inhibiting plasmid transfer (Zhang et al. 2025). Chlorine also downregulates *E. coli* porin OmpF, impairing molecular uptake and membrane transport. UV combined with low‐dose chlorination proves more effective than either alone. However, chlorine‐injured yet viable bacteria may exhibit increased plasmid transformation under certain conditions (Jin et al. 2020). At the molecular level, inhibitors targeting the conjugation machinery, such as bisphosphonates, intrabodies, and small molecules interfering with type IV secretion systems (T4SSs), have been shown to suppress horizontal gene transfer by disrupting relaxase activity, pilus formation, or DNA delivery (Swain and Sahoo 2025). Although these approaches demonstrate considerable potential for limiting ARG dissemination, their clinical applicability remains constrained by plasmid specificity, limited target range, and variable efficacy across bacterial species (Nash et al. 2012).

It is possible to effectively reverse bacterial resistance by eliminating plasmids through physical or chemical means. Some common physical methods for eliminating plasmids include heating, ultraviolet light irradiation (Pachnerová Brabcová et al. 2018), gamma ray irradiation (Popelářová et al. 2022), and microwave (MW) irradiation (Vrancianu et al. 2020; Ji et al. 2021). There have been more studies on methods of eliminating plasmids or eliminating the function of plasmids (plasmid curing) using chemical means. Disinfectants and oxidants such as ozone can effectively eliminate plasmids, and plant‐derived chemicals such as coumarin can block the replication of plasmids (Boy et al. 2022). Many DNA embedding agents, such as ethidium bromide and methyl orange, can also eliminate plasmids from various strains of bacteria (Huang et al. 2023). Other mechanisms include the caffeine‐dependent riboswitch to regulate the expression of the replication enzyme gene for the editing plasmid, thereby promoting the elimination of exogenous plasmids (Rodriguez‐Martinez et al. 2021). High concentrations of sodium dodecyl sulfate (SDS) can achieve the effect of removing resistance plasmids, but this can lead to gastrointestinal side effects. An emerging gene‐drive strategy, termed Dissemination Obstruction System (DoS), offers a novel approach for plasmid removal. By exploiting the inherent weakness of conjugative plasmids, engineered DoS plasmids can selectively infiltrate donor bacteria harboring target resistance plasmids, competitively eliminate them, and subsequently self‐destruct via built‐in suicide modules (Tsoi et al. 2025).

Class II adjuvants aim to prevent the dissemination of ARGs by inhibiting conjugative transfer or eliminating resistance plasmids. These strategies provide a preventive approach to AMR control, complementing conventional antibiotic and adjuvant therapies.

### 3.3 Class III—Modulating Host Defense to Enhance Antibiotic Efficacy

Against the backdrop of escalating antibiotic resistance, strategies that exploit the dynamic interplay between host, pathogen, and drug have gained attention. Harnessing the host's own defense mechanisms represents a novel means of enhancing antibiotic efficacy (Liu et al. 2021; Lu et al. 2025). This approach can be regarded as an “indirect” amplification of antibacterial activity: rather than directly inhibiting bacterial targets, these adjuvants act by activating or augmenting innate immune responses or by binding to bacterial membrane components such as LPS and phospholipids. In doing so, they disrupt membrane integrity or increase permeability, effectively “opening the door” for conventional antibiotics to exert their activity.

The innate immune system represents the first line of defense against infection. In macrophages, inducible antibacterial responses encompass a range of mechanisms, including LC3‐associated phagocytosis, metabolic reprogramming, lipid droplet formation, antimicrobial peptides, nutrient deprivation, metal ion toxicity, guanylate‐binding proteins, autophagy and nitric oxide production (Sweet et al. 2025). Similarly, stimulation of innate receptors has been shown to potentiate host defense; for example, the TLR2 agonist Pam3CSK4 enhances the antibacterial activity of GM‐CSF–induced neutrophils against methicillin‐resistant Staphylococcus aureus (Chen et al. 2019b). Beyond synthetic small molecules, plant‐derived natural products have also demonstrated immunomodulatory–antibiotic synergism. Polyphenols such as epigallocatechin gallate (EGCG), well known for their potent antioxidant properties, additionally exhibit anti‐inflammatory and antimicrobial activities (Ye et al. 2024). Collectively, these natural immunomodulators hold promise as adjuvants that “awaken” host defense pathways, indirectly reducing bacterial tolerance and thereby extending the therapeutic window for antibiotics. Antimicrobial peptides (AMPs) are widely distributed in nature, and their bactericidal mechanisms include membrane depolarization, pore formation and intracellular macromolecule aggregation. This disruption markedly enhances the permeability of hydrophobic antibiotics, such as rifampicin and erythromycin, as well as large glycopeptides like vancomycin (Zheng et al. 2025).

Class III adjuvants enhance antibiotic efficacy indirectly by activating host immune responses and improving bacterial clearance. By strengthening innate defenses, they offer a promising strategy to overcome infections caused by MDR pathogens.

Despite the considerable promise of natural immunomodulators and AMPs in enhancing antibiotic efficacy, several barriers still hinder their clinical translation. First, immunomodulators may trigger excessive inflammation or autoimmune responses, necessitating the development of precise delivery systems to achieve infection site–specific targeting. Second, AMPs suffer from poor in vivo stability and are prone to proteolytic degradation; however, their pharmacological properties can be improved through strategies such as D‐amino acid substitution, lipidation, or nanoparticle encapsulation.

## 4 New Technique Used to Reverse Bacterial Antibiotic Resistance

### 4.1 Gene Editing Technology Targeted Elimination of ARGs

Gene editing offers a powerful means to reverse antibiotic resistance by modifying bacterial genetic material. Among these approaches, CRISPR‐Cas systems have emerged as the most versatile tools. The CRISPR‐associated (Cas) family (Cas1–Cas14) encompasses enzymes with diverse functions, including nucleic acid cleavage, helicase, integrase, and polymerase activities (Koonin and Makarova 2019). Together with CRISPR sequences, these proteins form a conserved bacterial adaptive immune system that uses crRNA‐guided effector complexes to recognize and cleave target DNA or RNA, such as plasmids and phages (Zhang et al. 2021).

**Figure 5:**
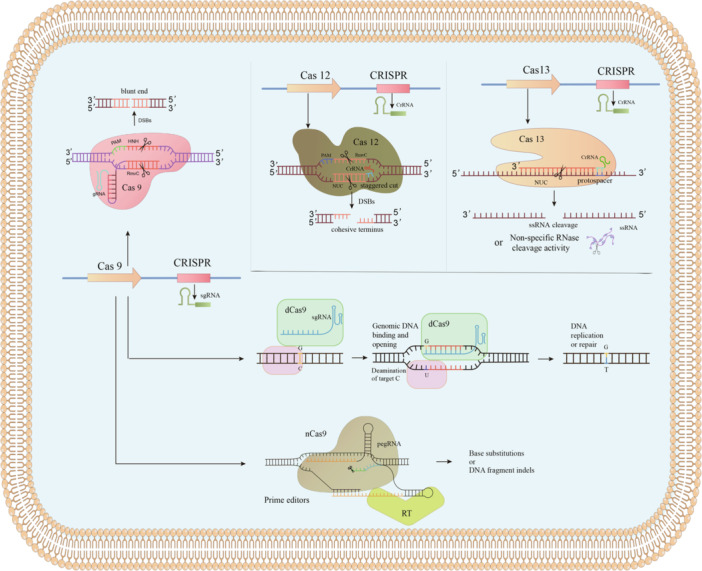
ARGs and ARB were eliminated by gene editing: CRISPR‐Cas9, Cas12, and Cas13 systems knock out antibiotic resistance genes or directly target and eliminate antibiotic‐resistant bacteria. Base editing terminates resistance gene expression early, while prime editing enables precise substitutions without inducing DNA double‐strand breaks (DSBs).

The CRISPR/Cas9 system, CRISPR‐Cas12 system, and CRISPR‐Cas13 system are extensively employed in bacteria to cleave double‐stranded DNA, single‐stranded DNA, and ssRNA invaders, effectively impeding the dissemination of ARB (Figure 5) (Karvelis et al. 2020). The Cas9 nuclease can be directed to any specific genomic locus through the use of a synthetic guide RNA (sgRNA) containing a 20‐nucleotide sequence at its 5’ end, which is capable of forming base pairs with the DNA in the genome. Following targeting, the Cas9 nuclease cleaves the genomic DNA using its enzymatic active site, resulting in a double‐strand break at the intended location within the genome. This process leads to either cell death or elimination of the targeted gene (Tao et al. 2023). The cutting mechanism of Cas12, unlike Cas9, results in the generation of sticky ends instead of blunt ends. This feature facilitates precise editing of DNA segments (Gao et al. 2020). Cas12a cuts both DNA and RNA, while compact Cas12b enables easier vector packaging and faster transfection (Karvelis et al. 2020). Cas12f1 is a programmable RNA‐guided dsDNA nuclease that recognizes and cuts dsDNA in a protospacer adjacent motif (PAM) dependent manner (Long et al. 2024). The delivery of the CRISPR‐Cas12f system to antimicrobial‐resistant strains can significantly improve curing efficiencies of the plasmid‐carried *mcr‐1* or *bla*_KPC_ genes (Su et al. 2024). CRISPR‐Cas13a is characterized by RNA‐guided single‐stranded RNA (ssRNA) cutting activity, which can limit the growth of host bacteria by degrading their RNA (Kim et al. 2025). CRISPR‐Cas13a can achieve sequence‐specific killing of carbapenem‐resistant *E. coli* and methicillin‐resistant *S. aureus* by targeting their corresponding ARGs (Kiga et al. 2020). CRISPR‐Cas systems mediated by enzymes such as Cas12a and Cas13 have emerged as innovative tools primarily used for the detection and monitoring of pathogenic bacteria.

CRISPR‐derived base editing enables precise single‐nucleotide modifications in genomic DNA while circumventing DNA double‐strand break (DSB) induction (Zhou et al. 2019). The current repertoire of DNA base editors includes two distinct types: the cytosine base editor (conversion of C‐G base pairs to T‐A base pairs), and the adenine base editor (conversion of A‐T base pairs to G‐C base pairs) (Diorio et al. 2022). Base editors typically consist of a defective Cas9 protein (Cas9D10A or Cas9D10AH840A) and a deaminase that fused with the Cas9 protein (Chen et al. 2018). Guided by the Cas9/sgRNA complex, deaminase can be directed to any genomic site, inactivating target genes by generating premature termination codons. In 2018, researchers successfully applied this technology in *S. aureus*, including MDR clinical strains, by fusing a catalytically inactive Cas9 (Cas9D10A) with cytidine deaminase (APOBEC1). By converting specific codons (CAA, CAG, CGA, TGG) in resistance genes to premature stop codons (TAA, TAG, TGA), they achieved rapid and efficient gene inactivation (Gu et al. 2018). Despite the fact that base editor technology has expanded the scope of gene editing therapy, it is still limited by the function of the deaminase domain, which cannot achieve arbitrary base exchanges and cannot insert or delete DNA sequences. By fusing cytidine deaminase with Cas9 nickase, researchers developed a highly efficient cytidine base editing system, pBECKP, which can achieve precise C → T transformation in chromosomes and plasmids. The pBECKP system can inactivate multiple resistance genes of *K. pneumoniae* by programmatically converting four codons (CAA, CAG, CGA, and TGG) into premature termination codons (Wang et al. 2018).

Prime editing (PE) is another unique gene editing technique that enables virtually any substitution in DNA without the need to produce a DSB (Chen and Liu 2023). Compared with traditional gene Editing techniques, PE has higher editing accuracy and specificity, and can effectively reduce the occurrence of unexpected mutations (Pacesa et al. 2024). PE has been widely applied in the genome editing of various eukaryotic cells, such as human cells, mouse cells and plant cells, etc. However, the application of this system in bacteria is still limited. Currently, PE has only been achieved in *E. coli* (Zhang et al. 2024a; Anzalone et al. 2020). A streamlined PE system tailored for the probiotic strain *E. coli* Nissle 1917 successfully enabled all major types of genome edits—deletion, insertion, and substitution—with efficiencies of 25.0%, 52.0%, and 66.7%, respectively (Chen et al. 2025b).

Efficient delivery of CRISPR components into bacteria remains a challenge to date. Currently employed methods, including nanoparticle, phage vectors, bacterial conjugative transfer, and so on. However, limitations in editing efficiency, off‐target effects, evaluation of long‐term antibacterial efficacy, and associated safety risks have significantly restricted the application of this technology in prokaryotic organisms. Developing endogenous CRISPR‐Cas systems that are widely distributed in bacteria and using the autonomously conjugative transfer method to achieve the delivery of this system is emerging as a new genomic editing strategy for the prevention and control of ARB (Zhou et al. 2023).

### 4.2 Photodynamic Therapy (PDT) to Overcome Multidrug Resistance

PDT represents an emerging strategy for overcoming MDR infections. PDT relies on the activation of photosensitizers by specific wavelengths of light, generating localized reactive oxygen species (ROS) that cause rapid membrane disruption, protein oxidation and nucleic acid damage. Recent studies demonstrate strong synergistic effects between PDT and conventional antibiotics, enabling re‐sensitization or enhanced susceptibility of resistant pathogens. Tanu et al. provided an updated overview of PDT‐antibiotic synergy and highlighted several photosensitizers that have advanced into preclinical evaluation (Tanu et al. 2025).

PDT causes extensive oxidation of membrane lipids, leading to increased permeability, loss of membrane potential, and compromised barrier function. This facilitates enhanced intracellular accumulation of antibiotics that normally suffer from poor penetration, such as aminoglycosides, fluoroquinolones, and β‐lactams. Several studies have shown that PDT pre‐treatment can reduce the MIC of these antibiotics by several folds in MDR pathogens (Zangirolami et al. 2024). PDT can effectively degrade extracellular polymeric substances (EPS), disrupts biofilm architecture and exposes previously shielded bacteria to antibiotics (Garcez et al. 2020). The combination of PDT and antibiotics has been systematically reviewed and recognized as a promising strategy to combat MDR and biofilm‐forming bacteria, by enhancing antibiotic uptake and overcoming resistance mechanisms (Ronqui et al. 2016).

Despite these promising effects, PDT still faces several limitations. The relatively short wavelength of activating light restricts its penetration into deep biological tissues (Idris et al. 2012), while the efficiency of ROS generation remains suboptimal and their diffusion distance is limited (Moan and Berg 1991). As an emerging technology, the antibacterial efficacy of PDT against resistant pathogens has attracted increasing attention. Consequently, combining PDT with complementary therapeutic modalities has become a major research focus to overcome these shortcomings and enhance antimicrobial performance. With the continuous advancement of nanotechnology, PDT can now be integrated with antibiotics, sonodynamic therapy, gas therapy, and nanozymes, significantly improving drug biosafety and the efficiency of biofilm disruption (Yu et al. 2024).

### 4.3 Potential of Nanotechnology in Antimicrobial Adjuvant Applications

**Figure 6:**
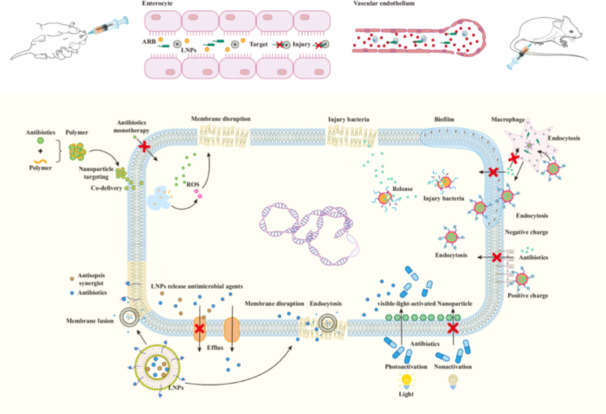
Nanoparticles in the treatment of iinfections caused by multidrug‐resistant organisms: Nanoparticles enhance antibiotic delivery in the intestine and bloodstream, improving therapeutic targeting. Antibiotics formulated as nanodrugs can disrupt biofilms, damage bacterial cell walls and membranes, and inhibit efflux pumps. Photoactive nanomaterials absorb near‐infrared light and convert it into heat to exert antibacterial effects.

Nanotechnology has demonstrated remarkable potential and advantages in antimicrobial therapy, particularly against ARB. Nanoparticles, with their unique physical, chemical, and biological properties, enable efficient delivery of antimicrobial agents in vivo. Moreover, they exhibit strong antibacterial activity‐either independently or synergistically with antibiotics, through mechanisms such as disrupting bacterial cell walls and membranes and effectively inhibiting efflux pumps (Figure 6). Photoactive nanomaterials absorb near‐infrared light (NIR) and convert it into heat, leading to cell membrane rupture, protein denaturation, and bacterial ablation. In addressing the challenge of antibiotic resistance in bacteria, nanotechnology presents a variety of strategies, including improving bioavailability, achieving precise targeted delivery, controlling drug release, employing stimulus‐responsive strategies, using biological vectors, and building cascades of targeted drug delivery systems. These innovative strategies not only significantly enhance treatment effectiveness, but also effectively slow the emergence and development of antibiotic resistance.

The presence of bacterial defense mechanisms such as efflux pumps and biofilms makes it difficult for antimicrobials to reach bacteria effectively, leading to multidrug resistance. The optimized antibiotic nanodelivery system can significantly improve the accumulation and penetration of antibiotics in bacterial cells, thereby improving the therapeutic index and bacterial sensitivity to antibiotics (Farhangi et al. 2018). The guanidinium‐functionalized cationic polymer pEt_20 effectively penetrates bacterial membranes and binds to various intracellular components. When self‐assembled with colistin, this structure enhances the colistin's affinity for resistant bacterial membranes, thereby reversing resistance in clinically isolated ARB (Zhao et al. 2023). PLGA nanoparticles loaded with ciprofloxacin enhance penetration through biofilm–mucus barriers, effectively combating *P. aeruginosa* pulmonary infections in cystic fibrosis. Similarly, xylitol‐loaded PLGA nanoparticles bind bacterial mannose residues to facilitate biofilm penetration and efficient drug release (Günday Türeli et al. 2017; Anjum et al. 2019). The anti‐tuberculosis drug rifampicin is loaded on silica nanoparticles and wrapped in outer membrane vesicles as a protective layer and targeting carrier, which can achieve accurate targeting and efficient intracellular uptake of the antimicrobial drug (Wu et al. 2021). I‐TPGS/Ga_2_O_3_ nanoparticles encapsulated Tigecycline for targeted treatment of Tigecycline resistant *K. pneumonia*. Tigacyclin‐containing nanoparticles prepared with pegylated vitamin E succinate (TPGS) can inhibit the activity of extracellular pump in drug‐resistant bacteria by decreasing the expression of efflux pump gene and transcriptional activator, and increase the concentration of antibiotics in bacterial cells (Kang et al. 2019). Using dimethylmaleic anhydride (DA) as a carrier and loaded with azithromycin (AZI), DA‐AZI, a bacterial microenvironment responsive nanoparticle that can clear bacterial membrane and inhibit its pathogenicity, was constructed by electrostatic composite technology. DA‐AZI nanoparticles can efficiently penetrate mucus and biofilm by charge inversion under acidic conditions, target entry into *P. aeruginosa* and increase its permeability to antibiotics (Li et al. 2024).

One of the major obstacles in developing antibiotics is that bacteria can quickly develop resistance, which requires a whole new research and clinical trial cycle to overcome. Nanoparticle technology provides a new approach to reversing bacterial resistance. On the one hand, nanoparticles can be loaded with non‐antibiotic drugs, which disrupt bacterial physiological processes through different mechanisms of action, thus restoring bacterial sensitivity to antibiotics. This strategy not only avoids the problem of antibiotic resistance of traditional antibiotics, but also expands the new thinking of antibacterial treatment. On the other hand, nanoparticles can also assemble reversal agents for antibiotic resistance with antibiotics, achieving optimal combination. This combination method can ensure effective accumulation and penetration of drugs in bacterial cells, while achieving the synergistic effects of the two drugs to improve treatment efficiency. By fine‐tuning the composition and structure of nanoparticles, the release characteristics and targeting of drugs can be further optimized, providing strong support for precision medicine. The self‐assembly synthesis of berberine (BBR) and chlorogenic acid (CGA) results in the formation of supramolecular herbal nanoparticles, which, when combined with ampicillin, can produce a synergistic inhibitory effect against MRSA (Fu et al. 2024). The liposome co‐delivery system Lipo‐cc, which encapsulates curcumin and colistin, allows the antibiotic and antibacterial adjuvant to reach the infected site at an effective dose simultaneously, thereby achieving the best therapeutic effect (Qin et al. 2023). Purple saponin nanoparticles are loaded with clavulanic acid (β‐lactamase inhibitor) and amoxicillin (β‐lactam antibiotic), and coated with cationic surfactant octadecyl trimethyl ammonium bromide (ODTAB) through electrostatic attraction to bind to the bacterial cell wall and enhance local drug concentration. These nanoparticles enable the highly efficient binding of β‐lactamase inhibitor to the β‐lactamase produced within the cell, thereby reversing bacterial resistance to antibiotics (Filby et al. 2022).

Photocatalytic nanomaterials can be activated by light, showing the ability to reverse drug resistance. By activating with non‐lethal visible light, synthetic molecular nanomachines (MNMs) significantly enhance the antibacterial activity of various traditional antibiotics against Gram‐negative bacteria, even those antibiotics that are usually only effective against Gram‐positive bacteria, while reducing the required antibiotic concentration for killing. These MNMs can bind to negatively charged phospholipids on the bacterial inner membrane, resulting in an increase in the permeability of the cell membrane. After being activated by light, the MNMs undergo unidirectional rotation, generating mechanical force that pushes the molecules through the cell membrane, thereby inhibiting the function of efflux pumps. The synergistic effects of MNMs on cell membrane permeability and efflux pump function accelerate the accumulation of antibiotics inside the cell, thereby achieving their powerful antibacterial effects (Santos et al. 2024). Bionic liposomes loaded with infrared photosensitizer black phosphorus quantum dots (BPQDs) and antibiotic Amikacin (AM) (AB@LRM) are a delivery system made by enclosing a mixed membrane shell of red blood cells and macrophages. The mixed membrane shell of red blood cells and macrophages enables the nanoparticles to accurately target bacteria while circulating in the internal environment for a long time, and the use of photosensitizing agents can effectively increase the sensitivity of bacteria to antibiotics (Liu et al. 2024b).

Bacterial cell envelope, biofilm protection and macrophage shelter can significantly enhance bacterial resistance to antibiotics. However, the electrostatic action of nanoparticles provides a new way to solve this problem. The polycationic micelle system PP‐PEI, based on polylactide‐PEG2K‐PEI2K copolymer (PLA5K‐PEG2K‐PEI2K), is used to load tetracycline. In this system, a unique penetration mechanism is formed between the positively charged PEI blocks and the negatively charged components in the biological barrier through non‐specific electrostatic interaction, which enables the PP‐PEI system to efficiently penetrate the biological barrier and achieve significant bactericidal effect against MDR gram‐negative bacteria (Guo et al. 2020). Other similar studies have demonstrated the antibacterial potential of positively charged metal oxide nanoparticles (CeO‐NPs). These nanoparticles can bind tightly to the bacterial cell membrane and enhance the permeability of the cell membrane through interaction with the bacterial cell outer membrane, so that antibiotics can enter the bacterial interior smoothly, thus significantly enhancing antibacterial activity (Bellio et al. 2018). In addition, glycol chitosan (GCS) coated ciprofloxacin (CIP)—supported asymmetric CaCO_3_ nanoparticles (CC@GCs) also demonstrated a unique antibacterial mechanism. In acidic environments, such as those caused by bacterial infection, the CO_2_ pressure generated by the reaction of CaCO_3_ with acid can promote the migration of nanoparticles and increase their chances of contact with bacteria. Through electrostatic bonding, the nanoparticles are able to adhere tightly to the bacteria, increasing the concentration of drugs near the bacteria (Jiang et al. 2023). Another study encapsulated imipenem into biodegradable polyƐ‐caprolactone (PCL) and polylactide‐ethyl ester copolymer (PLGA) nanocapsules. This encapsulation method not only overcomes the adhesion and transmission of microorganisms, but also protects imipenem from enzymatic degradation of resistant isolates, thus effectively killing imipenem resistant bacteria. At the same time, the nanoparticles also showed good mutation inhibition (Shaaban et al. 2017).

### 4.4 Microbial Interactions Reverse ARB Infections

**Figure 7:**
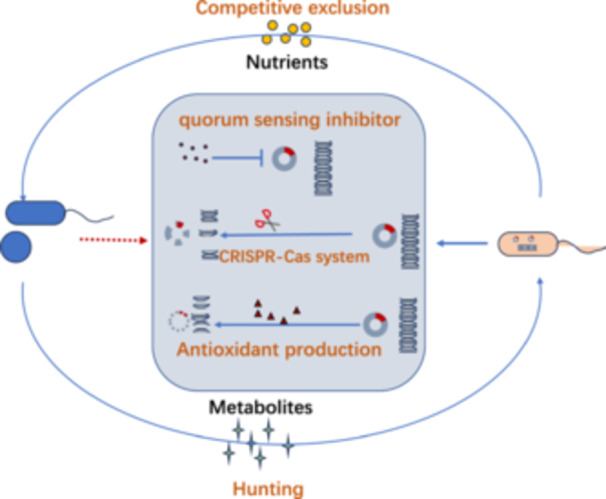
Microbial interactions inhibit antibiotic‐resistant bacteria: Probiotics suppress resistant strains by competing for ecological niches or releasing inhibitory metabolites, while other microbes block ARGs transmission by interfering with quorum sensing or degrading ARGs.

Ecological competition mechanisms can effectively constrain the evolution of antibiotic resistance (Letten et al. 2021). Probiotics establish microbial consortia with ecological dominance in specific niches, exerting multidimensional suppression on resistant strains through competitive exclusion, quorum sensing (QS) interference, antimicrobial metabolite secretion, and host immune modulation (Figure 7). This ecological intervention not only diminishes resistant pathogens' colonization advantage but crucially reshapes their population selection pressure, potentially restoring antimicrobial susceptibility and achieving resistance reversal at the microbiome level (Li et al. 2020).

Probiotics regulate intestinal ecology through competitive exclusion of resistant pathogens, thereby reducing their gastrointestinal colonization. As major reservoirs of antibiotic resistance genes (ARGs), gut microbiomes facilitate horizontal gene transfer to pathogens. Probiotics demonstrate host‐specific and antibiotic‐dependent modulation of ARG dynamics (Su et al. 2022). QS‐mediated regulation of biofilm formation, efflux pumps, and secretion systems constitutes critical resistance mechanisms (Salman et al. 2023). Probiotic‐derived metabolites, exemplified by bacillomycin D from *Bacillus subtilis* competing with Staphylococcus aureus autoinducing peptides (AIPs), effectively block QS signaling and inhibit nasal colonization by MRSA (Piewngam et al. 2018).

Biofilm formation significantly reduces antimicrobial susceptibility. *Lactiplantibacillus plantarum* and *Lacticaseibacillus rhamnosus* suppress *Streptococcus pyogenes* biofilms (Gómez‐Mejia et al. 2024), while *Lactobacillus acidophilus*, *Lactobacillus plantarum* and *Streptococcus salivarius* compromise *S. aureus* biofilm viability via diffusible non‐acidic factors (Humphreys and McBain 2019). Optimized probiotic consortia (*L. rhamnosus*: *L. sakei*: *B. subtilis* = 5: 5: 1) inhibit *K. pneumoniae* biofilm‐associated gene expression through organic acid and cyclic di‐GMP signaling (Zhang et al. 2024b). Direct ARG modulation occurs through mechanisms like short‐chain fatty acids from *Lacticaseibacillus casei* suppressing ARG expression (Yao et al. 2021). Synergistic approaches utilizing *Lactobacillus*‐synthesized C1‐AuNP/C1‐AgNP nanoparticles enhance biofilm inhibition and eradication through improved biocompatibility (Kang et al. 2023).

Despite therapeutic potential, probiotic efficacy exhibits interindividual variability. Safety concerns persist regarding probiotic‐derived ARG dissemination via horizontal gene transfer and opportunistic infections in immunocompromised hosts (Crits‐Christoph et al. 2022). Unintended microbiome perturbations and insufficient clinical evidence necessitate further mechanistic studies to establish standardized therapeutic protocols.

## 5 Conclusions

The extensive use of antibiotics has imposed significant evolutionary pressures on bacteria, leading to widespread resistance and ultimately rendering antibiotics ineffective. The development of new antibiotics faces theoretical and clinical challenges, further exacerbating the crisis of antibiotic resistance. Therefore, research based on the evolutionary characteristics of bacterial antibiotic resistance and the concept of reversing resistance has become a forward‐looking paradigm for preventing and controlling the spread of resistant bacteria. By delving into the genetic basis and mechanisms of bacterial resistance, researchers can develop innovative therapeutic strategies to restore the effectiveness of antibiotics in a highly targeted manner. The application of antibiotic adjuvants and the development of resistance reversal methods not only accurately restore the antibacterial efficacy of existing antibiotics but also effectively curb the emergence and evolution of resistance, laying a solid foundation for the continued use of antibiotics. As the limitations of traditional antibiotic therapy become increasingly evident, the development of nanotechnology and microbial technologies offers new solutions to this challenge. Future tasks still require further integration and development of innovative technologies, and continuous optimization of methods and strategies based on existing foundations.

## Author Contributions

**Tianjiao Li:** data curation (equal), investigation (equal), writing – original draft (lead). **Fei Zeng:** data curation (equal), writing – original draft (equal). **Jie Zhang:** data curation (equal). **Yuangong Zhang:** writing – review and editing (equal). **Wenjuan Yin:** data curation (equal), investigation (equal), writing – review and editing (lead).

## Ethics Statement

The authors have nothing to report.

## Conflicts of Interest

The authors declare no conflicts of interest.
